# A Case Report: Unveiling the Underlying Cause of Recurrent Pericarditis in a Patient With Undifferentiated Connective Tissue Disease

**DOI:** 10.7759/cureus.54113

**Published:** 2024-02-13

**Authors:** Samantha Boever, Kareem Bannis

**Affiliations:** 1 Internal Medicine, A.T. Still University School of Osteopathic Medicine, Mesa, USA; 2 Internal Medicine, Adelante Healthcare, Phoenix, USA

**Keywords:** treatment resistant pericarditis, rheumatoid arthriitis, undifferentiated connective tissue disease, recurrent pericarditis, autoimmune pericarditis

## Abstract

Undifferentiated connective tissue disease (UCTD) is a condition characterized by symptoms and laboratory findings related to various systematic autoimmune diseases. Severe symptoms like chest pain in patients with UCTD could suggest an underlying secondary condition, such as pericarditis. Our case involves a 36-year-old woman with a history of UCTD and recently diagnosed rheumatoid arthritis (RA) who presented with persistent sub-sternal chest pain and pressure that began three weeks ago. Over the past year, she experienced six similar episodes of chest pain, diagnosed as idiopathic pericarditis. She promptly underwent treatment with oral prednisone and was instructed to continue her current medications (colchicine, methotrexate, and Plaquenil). Subsequent laboratory results, obtained several days posttreatment, revealed an elevated C-reactive protein (CRP), normal erythrocyte sedimentation rate (ESR), an elevated rheumatoid factor, and a normal echocardiogram, suggesting resolution of the acute flare. Despite having a comprehensive treatment regimen, the patient continues to experience recurrent pericarditis episodes. The cause of the recurrence remains uncertain, potentially associated with repeated use of high-dose steroids and a recent diagnosis of RA. Consequently, her rheumatologist opted to initiate treatment with intravenous Golimumab to better manage the RA and potentially address recurrent pericarditis. Physicians should maintain a heightened clinical suspicion of pericarditis in UCTD patients experiencing chest pain, as initiating prompt treatment helps prevent long-term complications and can be lifesaving in certain instances.

## Introduction

The prevalence of cardiac involvement is a notable aspect among patients diagnosed with connective tissue and rheumatic diseases, warranting comprehensive investigation and prompt treatment [1]. Affected structures commonly include the pericardium (resulting in pericarditis and pericardial effusion), myocardium (leading to myocarditis, cardiomyopathy, and congestive heart failure), coronary arteries (associated with acute coronary syndromes), endocardium (involving valve issues and thrombosis), and vascular structures (contributing to aneurysm formation) [1]. Notably, approximately 2% to 7% of cases of acute pericarditis and 10% of cases of recurrent pericarditis are attributed to rheumatic causes. In a study published in 2007, which followed 453 patients with acute pericarditis prospectively from 1996 to 2004, findings revealed a systemic autoimmune cause for pericarditis in 33 of the total patients [2].

The development of acute pericarditis in patients with autoimmune conditions is typically attributed to the induction of an inflammatory state, specifically through antibodies and reactive T cells. Common diseases in this category include systemic lupus erythematosus (SLE), rheumatoid arthritis (RA), Sjogren's syndrome, polymyositis/dermatomyositis, scleroderma, and vasculitis [3]. Patients meeting the criteria for multiple autoimmune diseases may receive a diagnosis of connective tissue disease (CTD) and, as a result, are at a heightened risk of experiencing recurrent episodes of pericarditis. 

CTDs pose a considerable challenge to diagnose due to their inherent complexity and multifactorial nature. Currently, criteria exist for categorizing five autoimmune CTDs that include scleroderma, myositis, Sjogren’s syndrome, RA, and SLE [4]. The primary guiding factors for diagnosis involve specific clinical signs, symptoms, and the presence of autoantibodies. In addition, diagnostic challenges often occur due to overlapping clinical manifestations in the early stages of autoimmune diseases. However, as the disease progresses, rheumatologists typically manage to make a clinical diagnosis. 

Historically, CTD serves as an umbrella term describing a group of diseases characterized by inflammatory autoimmune processes affecting various organ systems. Patients frequently exhibit clinical signs and symptoms indicative of autoimmune disorders; however, they often do not fully meet the necessary criteria to diagnose a specific CTD. For instance, a patient might display a constellation of symptoms including Raynaud's syndrome, a positive antinuclear antibody (ANA), and arthralgias/myalgias. While all the aforementioned symptoms are commonly associated with autoimmune disease, the manifestations alone fail to meet the criteria for a defined CTD such as SLE. In cases where the diagnosis remains unclear, undifferentiated CTD (UCTD) is a viable diagnosis [5].

A definition for UCTD has not been published by either the American College of Rheumatology or the European Alliance of Association for Rheumatology. The widely accepted definition of UCTD stipulates that a patient must exhibit a positive ANA measured on two separate occasions along with at least one clinical symptom of a CTD while still failing to meet the established criteria for a CTD [5]. Consequently, the management and treatment of this disease varies and lacks specific guidelines. Management primarily relies on patient-reported symptoms, such as the use of non-steroidal anti-inflammatory drugs (NSAIDs) for arthralgia. Patients who report more concerning symptoms, such as chest pain, require immediate evaluation and treatment due to the heightened risk of developing pericarditis. We present a unique case in which a patient with a history of UCTD experienced multiple episodes of pericarditis that resolved with immediate treatment. The patient has been informed and has consented to the documentation of this case report.

## Case presentation

**Figure 1:**
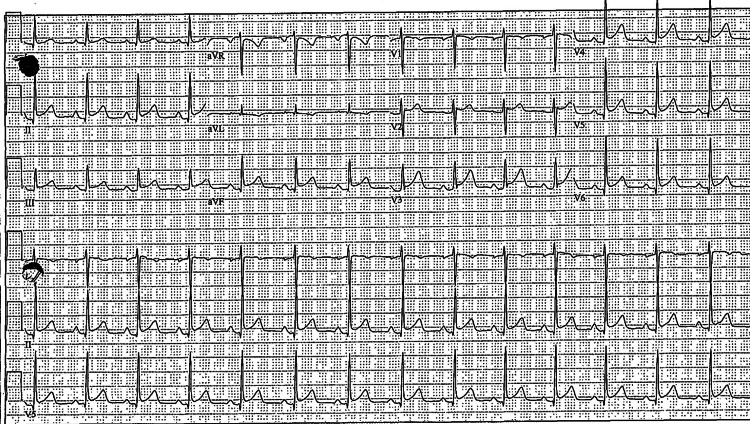
Electrocardiogram showing diffuse ST segment elevations, PR depressions, and early repolarizations.

Our patient is a 36-year-old woman who presented to her primary care physician (PCP) with a three-week history of substernal chest pain and pressure. She described the pain as constant and non-radiating, exacerbated by coughing and relieved by sitting upright and leaning forward. Associated symptoms included shortness of breath and exertional dyspnea. The patient reported a history of similar episodes of chest pain, all of which had been diagnosed as idiopathic pericarditis flares over the last three to four years. Previous instances were confirmed through electrocardiogram (EKG) findings consistent with pericarditis (Figure 1), clinical symptoms (chest pain that improved with sitting up and leaning forward), and physical exam findings (pericardial friction rub heard on auscultation). In the last year alone, she experienced six episodes successfully treated with oral (PO) prednisone 20 mg, once daily for five days. She is currently taking colchicine 0.6 mg PO twice daily (BID), and Aleve as needed. Previous attempts with Azathioprine and CellCept were discontinued due to gastrointestinal side effects and fatigue, respectively. 

The patient had a complicated past medical history. At the age of 12, she developed uveitis, knee pain, and bursitis, which were evaluated by Pediatric Rheumatology without receiving a specific diagnosis. Her uveitis was managed with NSAIDs. By age 20, she had continued to experience joint pain and had developed dry eyes, leading to a diagnosis of UCTD. She had been on Plaquenil since that time.

Additionally, she reported an erythematous rash on her right cheek exacerbated by sun exposure, labeled as *lupus tumidus* based on biopsy results. Plaquenil provides moderate relief for the facial rash. She tested positive for ANA and antiphospholipid antibodies in the past but never received an official diagnosis of SLE. She had no history of deep vein thrombosis or pulmonary embolism and reported symptoms such as photosensitivity, hair thinning, and oral sores. 

She was previously followed by a pulmonologist due to reactive airway disease and pneumonitis. The origin of her pneumonitis remains uncertain but may be linked to her UCTD or previous occupational exposure to fungi. 

The patient suffered from chronic low back and hip pain exacerbated by movement. Previous magnetic resonance imaging (MRI) revealed bilateral hip labral tears, for which she received steroid injections. She denied experiencing leg pain or swelling and reported that her knee pain worsened with walking. She used hydrocodone to manage her pain and had been advised to avoid NSAIDs due to a previously developed gastric ulcer and a prior left nephrectomy resulting from a ureteropelvic junction obstruction.

Notably, one year ago, she received a diagnosis of RA, characterized by a positive rheumatoid factor and a negative ANA. Since then, she underwent treatment with methotrexate.

On physical examination, she was tachycardic and tachypneic. A pericardial friction rub was detected over the left sternal border on cardiac auscultation. Lung sounds were clear bilaterally, without any wheezing, rhonchi, or rales. Her abdominal examination was unremarkable. Examination of the extremities showed no signs of cyanosis, clubbing, or edema.

Due to a high clinical suspicion of pericarditis stemming from the patient's history of recurrent episodes, treatment was initiated without delay. She was instructed to take prednisone at a dosage of 20 mg PO once daily for five days, followed by 10 mg for the subsequent seven days, and a further reduction to 5 mg for the following seven days, ultimately leading to discontinuation. 

**Figure 2:**
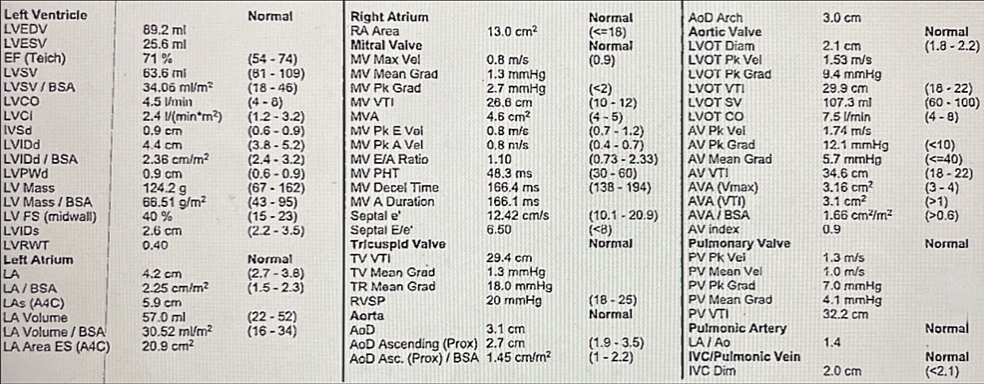
Transthoracic echocardiogram showing no acute abnormalities.

Several days after initiating treatment with prednisone, pertinent laboratory results revealed an elevated C-reactive protein, normal erythrocyte sedimentation rate, and an elevated rheumatoid factor (Table 1). The results of her thyroid panel, comprehensive metabolic panel (CMP), and complete blood count (CBC) were unremarkable. Additionally, her transthoracic echocardiogram (TTE) demonstrated normal findings, including a visible ejection fraction ranging from 50% to 55%, trace regurgitation in both the mitral and tricuspid valves, and the absence of pericardial effusion (Figure 2). These findings suggested a likely resolution of the acute flare.

**Table 1:** Laboratory findings. IU, international units

Test	Result	Reference range
C-reactive protein	7.5 mg/L	<4.9 mg/L
Erythrocyte sedimentation rate	3.0 mm/hour	<20 mm/hour
Rheumatoid factor	19.0 IU/mL	<3.5 IU/mL

Due to the persistent recurrence of pericarditis episodes in this patient, her cardiologist recommended that she continue her current colchicine dose (0.6 mg PO, twice daily [BID]) for an additional six months, with the intent to gradually taper and discontinue the medication thereafter. Additionally, to promote diuresis and mitigate her acute pericardial flares, she was advised to discontinue Losartan 100 mg and initiate Losartan Potassium-Hydrochlorothiazide 50-12 mg tablets PO, once daily. Due to the unclear origin of her recurring flares, possibly associated with frequent use of moderate- to high-dose steroids or her recent diagnosis of RA, her rheumatologist elected to initiate treatment with intravenous infusions of Golimumab, a tumor necrosis factor-alpha (TNF-α) antagonist. Golimumab was administered alongside her existing treatment plan, which included injectable methotrexate at a dosage of 10 mg/0.4 mL. Finally, she was instructed to continue her ongoing treatment regimen for UCTD, which included Plaquenil 200 mg PO, BID.

## Discussion

Clinical suspicion for pericarditis should be elevated in patients who complain of chest pain and have a history of rheumatic conditions. The presence of systemic symptoms such as fever, hypotension, increased jugular vein pressure, and immunocompromised status should increase concern. Pericarditis flares in the context of rheumatologic and CTDs increase the risk of developing complications, including cardiac tamponade and constrictive pericarditis [3]. Failure to detect and promptly treat pericarditis flares increases morbidity and mortality. 

The management of recurrent pericarditis poses challenges due to the absence of a clearly defined treatment algorithm. Approaches vary based on patient response and clinical manifestations. Colchicine is commonly employed as a first-line agent, administered at a dose of 0.5-0.6 mg twice daily for three months. Alternatively, NSAIDs, including ibuprofen 400-800 mg are sometimes administered three times daily for four weeks to manage episodes of recurrent pericarditis [3]. Second-line agents such as prednisone, prescribed at a dose of 0.2-0.5 mg/kg, are considered, but caution is exercised due to increased recurrence rates with the use of moderate to high doses [6]. As exhibited in the case, short courses of prednisone ranging from 20 to 40 mg for two weeks are generally sufficient to control acute pericarditis flares. 

While colchicine, NSAIDs, and steroids are effective in mitigating acute pericarditis flares, the focus of treatment for patients with recurrent episodes should address the underlying causes (autoimmune or CTD). Methotrexate, often used at doses up to 20 mg weekly, is a frequent choice in treating autoimmune diseases. Other nonbiological agents such as Azathioprine and Mycophenolate are also commonly used in the treatment of various autoimmune conditions. Additionally, emerging research suggests the use of immunosuppressive biological agents (i.e., interleukin antagonists) as alternative options to the use of steroids for pericarditis flares [3].

Significantly, the patient in this case began experiencing an increased frequency of recurrent pericarditis episodes shortly after being diagnosed with RA. Since her diagnosis in 2023, she has encountered a total of six episodes. This observation aligns with current research indicating that pericardial disease is the predominant cardiac manifestation linked to RA, affecting approximately one-third of RA patients [3,7]. Furthermore, the onset of cardiac involvement within the first year of an RA diagnosis is associated with elevated mortality rates. Notably, pericardial involvement is more common in patients not utilizing disease-modifying antirheumatic drugs (i.e., methotrexate) [7].

Furthermore, while the patient was never officially diagnosed with SLE, she exhibited features associated with the condition, such as a previously positive ANA and antiphospholipid antibody test, along with the presence of a malar rash (lupus tumidus), which could have contributed to her recurring pericarditis episodes. The pathogenesis of SLE involves autoantibodies and immune complexes, contributing to heightened inflammation and the activation of the complement pathway. Notably, immune complexes have been detected in the pericardial fluid of SLE patients [8]. Autopsy findings in SLE cases reveal immunoglobulin deposition and complement components within pericardial vessel walls, accompanied by inflammatory cell infiltration [9].

Despite diligent adherence to her comprehensive treatment regimen which includes methotrexate, Plaquenil, colchicine, and prednisone for acute flares, the patient in this case continues to experience recurrent episodes of pericarditis. Emerging research indicates a potential correlation between the frequent administration of high-dose steroids and heightened recurrence rates of acute pericardial flares [6]. Furthermore, the prolonged use of steroids raises concerns about potential side effects, including osteoporosis, hypertension, peptic ulcers, metabolic syndrome, and Cushingoid symptoms [10]. Nevertheless, the use of high-dose steroids during acute flares affords the patient effective symptom management, eliminating the necessity for hospitalization. 

## Conclusions

In the presented case, the patient underwent a treatment regimen involving methotrexate for RA, Plaquenil for symptoms associated with lupus, and a combination of colchicine and prednisone to address pericarditis flares. Despite the implementation of a comprehensive treatment plan, she continued to experience recurring episodes of pericarditis of unknown origin. Given the persistence of her symptoms and heightened suspicion of RA as a contributing factor, her rheumatologist initiated intravenous infusions of Golimumab, a TNF-α inhibitor currently used in the treatment of RA. Should the recurrent flares of pericarditis originate from her underlying RA, the administration of Golimumab is anticipated to aid in the prevention of future episodes. 
